# ICH S1 prospective evaluation study: weight of evidence approach to predict outcome and value of 2-year rat carcinogenicity studies. A report from the regulatory authorities subgroup

**DOI:** 10.3389/ftox.2024.1353783

**Published:** 2024-04-11

**Authors:** Todd Bourcier, Timothy McGovern, Tania Cavaliero, Geoffrey Ebere, Akiyoshi Nishikawa, Jihei Nishimura, Kumiko Ogawa, Markku Pasanen, Alisa Vespa, Jan Willem Van der Laan

**Affiliations:** ^1^ Food and Drug Administration, Silver Spring, MD, United States; ^2^ Swissmedic, Swiss Agency for Therapeutic Products, Bern, Switzerland; ^3^ Health Canada, Pharmaceutical Drugs Directorate, Ottawa, ON, Canada; ^4^ National Institute of Health Sciences, Kanagawa, Japan; ^5^ Pharmaceuticals and Medical Devices Agency, Tokyo, Japan; ^6^ Faculty of Health Sciences, School of Pharmacy, University of Eastern Finland, Kuopio, Finland; ^7^ Medicines Evaluation Board, Utrecht, Netherlands

**Keywords:** carcinogenicity, bioassay, weight of evidence, ICH S1B(R1), risk assessment, pharmaceuticals, 3R principles

## Abstract

**Introduction:** The International Council for Harmonisation of Technical Requirements for Pharmaceuticals for Human Use (ICH) initiated a process in 2012 to revise the S1B Guideline “Testing for Carcinogenicity of Pharmaceuticals”. Previous retrospective analysis indicated the importance of histopathological risk factors in chronic toxicity studies, evidence of endocrine perturbation, and positive genetic toxicology results as potentially predictive indicators of carcinogenic risk. In addition, a relationship between pharmacodynamic activity and carcinogenicity outcome in long-term rodent studies has been reported. It was postulated that these factors could be evaluated in a Weight-of-Evidence (WoE) approach to predict the outcome of a 2-year rat study.

**Methods:** The ICH S1B(R1) Expert Working Group (EWG) conducted a Prospective Evaluation Study (PES) to determine the regulatory feasibility of this WoE approach. Drug Regulatory Authorities (DRAs) evaluated 49 Carcinogenicity Assessment Documents (CADs), which describe the WoE for submitted pharmaceutical compounds. Each compound was categorized into a carcinogenic risk category including a statement of the value of the 2-year rat study. The outcome of the completed 2-year rat studies was evaluated in relation to the prospective CAD to determine the accuracy of predictions.

**Results:** Based on the results of the PES, the EWG concluded that the evaluation process for assessing human carcinogenic risk of pharmaceuticals described in ICH S1B could be expanded to include a WoE approach. Approximately 27% of 2-year rat studies could be avoided in cases where DRAs and sponsors unanimously agreed that such a study would not add value.

**Discussion:** Key factors supporting a WoE assessment were identified: data that inform carcinogenic potential based on drug target biology and the primary pharmacologic mechanism of the parent compound and major human metabolites; results from secondary pharmacology screens for this compound and major human metabolites that inform carcinogenic risk; histopathology data from repeated-dose toxicity studies; evidence for hormonal perturbation; genotoxicity data; and evidence of immune modulation. The outcome of the PES indicates that a WoE approach can be used in place of conducting a 2-year rat study for some pharmaceuticals. These data were used by the ICH S1B(R1) EWG to write the R1 Addendum to the S1B Guideline published in August 2022.

## Introduction

The International Council for Harmonisation of Technical Requirements for Pharmaceuticals for Human Use (ICH) is a key international organization involving regulators and industry that develops internationally harmonized scientific and technical guidelines to support global licensing of human medicines. The ICH S1B guideline “Testing for Carcinogenicity of Pharmaceuticals” provides recommendations on approaches for evaluating the carcinogenic potential of pharmaceuticals which can include the conduct of a 2-year rat carcinogenicity study. An Addendum to the ICH S1B guideline was recently introduced to include a Weight of Evidence (WoE) approach (ICH S1B(R1), 2022) which involves an assessment of WoE factors to inform whether a 2-year rat carcinogenicity study adds value to the assessment of human carcinogenic risk. The recommendations outlined in the Addendum are in part based on the outcome of a Prospective Evaluation Study (PES) conducted under ICH S1(R1) Proposed Change to Rodent Carcinogenicity Testing of Pharmaceuticals–Regulatory Notice Document (ICH, 2013) between 2013–2020.

The primary impetus for updating the guidance was the retrospective analysis of a dataset of 182 blinded compounds from 13 PhRMA companies and a further dataset of 76 IARC Class 1 and 2A compounds (Sistare et al., 2011) which indicated that the absence of (1) histopathologic risk factors for rat neoplasia in chronic toxicology studies, (2) evidence of hormonal perturbation or intended endocrine pharmacology, and (3) positive genetic toxicology results predicted a negative tumor outcome in 82% of 2-year rat carcinogenicity studies evaluated. The rat tumor findings in the remaining 18% of compounds were judged to be of questionable human relevance. It was proposed that compounds meeting these criteria would have a low likelihood of being rat carcinogens and therefore an adequate assessment of human carcinogenic risk could be based on these criteria and completed without results from a 2-year rat study.

Furthermore, a retrospective analysis of a dataset of 255 unblinded compounds from industry and regulatory agencies showed a relationship between pharmacodynamic activity and histopathology findings in rats after 6 months of treatment and subsequently with carcinogenicity outcome in the 2-year rat study (Van der Laan et al., 2016a). Both a positive and a negative relationship was observed and indicated that a more complete knowledge of drug target pharmacology may contribute to the improved prediction of carcinogenicity outcome in the 2-year rat study (Van der Laan et al., 2016a). In another dataset of 289 human pharmaceuticals, the ability to predict rat non-carcinogens based on pharmacology and histopathology had a success rate of 92% whereas the ability to predict rat carcinogens was 98% (Van der Laan et al., 2016b).

These retrospective analyses supported the hypothesis put forward by the ICH S1B(R1) expert working group (EWG) in a Regulatory Notice Document (ICH, 2013). That is, knowledge of pharmacologic target(s) and signaling pathway(s), together with toxicological data, is sufficient to characterize the carcinogenic potential of a pharmaceutical and therefore sufficient to determine whether the conduct of a 2-year rat study would add value to the assessment of human carcinogenic risk. Prospective studies had not been conducted to discern the predictivity of a WoE approach that includes information on drug target pharmacology together with compound-specific toxicology to assess the outcome of a 2-year rat study and its relation to assessing human carcinogenic risk. Moreover, there was no information that addressed if Drug Regulatory Authorities (DRAs) and industry could align on reasonably consistent safety and regulatory decisions based on the conclusion of a WoE assessment, and in regard to the need for a 2-year rat bioassay in assessing human carcinogenic risk.

A PES was therefore conducted to determine the regulatory feasibility of this WoE approach and conclusions from these retrospective analyses in a real-world setting, where prior knowledge of the 2-year rat carcinogenicity study outcome is not available. The specific objectives of the PES were as follows:

• To determine if the WoE approach is sufficiently robust to predict the outcome and value of a 2-year rat carcinogenicity study,
• To define the specific factors that contribute to a WoE assessment leading to a conclusion that a 2-year rat study does, or does not, contribute to the assessment of human carcinogenic risk,
• To assess concordance of predictions and statements of value among DRAs and between DRAs and pharmaceutical sponsors.

## Methods

**FIGURE 1:**
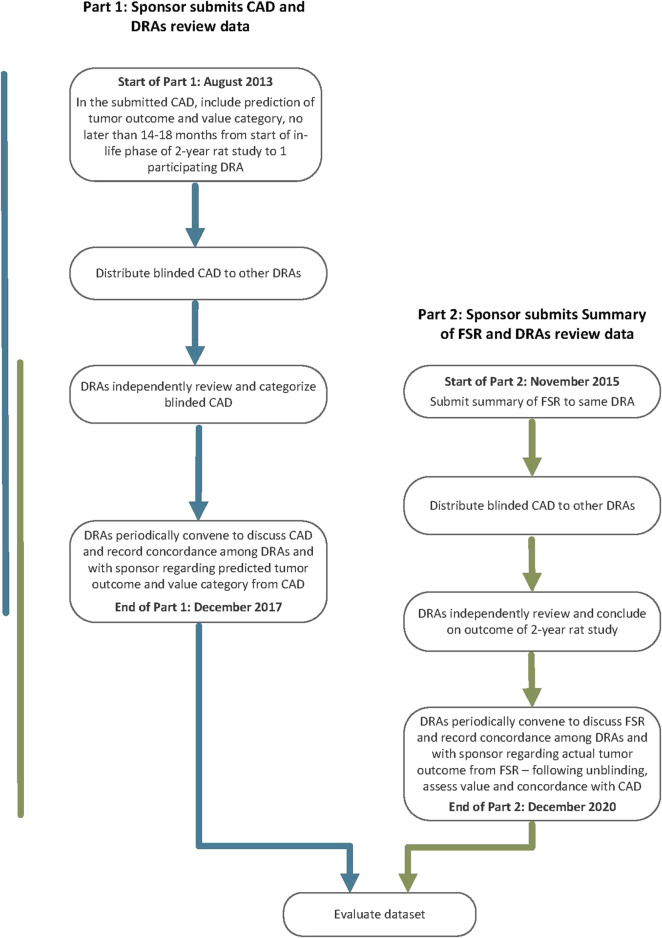
Flow chart outlining the design of the Prospective Evaluation Study.

The PES called for sponsors to conduct a prospective assessment addressing human carcinogenic risk of a pharmaceutical under active development and the anticipated outcome and value of a 2-year rat study to that assessment using specific WoE criteria (ICH, 2013). The assessment, referred to as a Carcinogenicity Assessment Document or CAD, was submitted to one of the five participating DRAs (Figure 1: Part 1). The outcome of the prospective assessment was then compared with the outcome of the 2-year rat study (Figure 1: Part 2). Therefore, following completion of the 2-year rat study, a summary of the final study report (FSR) was submitted to the same DRA receiving the CAD submission. After completion of Part 1 and Part 2 of the PES, WoE criteria addressed in the CADs were re-evaluated for the dataset regarding the value for predicting tumor outcome and assessing overall human carcinogenic risk.

### Carcinogenicity Assessment Documents

Participating sponsors submitting a CAD were requested to address specific WoE factors considered pertinent to the assessment of carcinogenic potential (ICH, 2013). Based on the level of certainty regarding carcinogenicity risk and its potential human relevance, sponsors were requested to include a prediction of tumor outcome from the planned or ongoing 2-year rat study and assign the pharmaceutical to one of 4 carcinogenicity risk categories described in Table 1. The sponsor was also requested to state the projected value of the rat carcinogenicity study outcome.

**TABLE 1:** Carcinogenicity risk categories.

Category 1	Highly likely to be tumorigenic in humans such that a 2-year rat, 2-year mouse, or transgenic mouse carcinogenicity studies would not add value.
Category 2	Tumorigenic potential for humans is uncertain and rodent carcinogenicity studies are likely to add value to human risk assessment.
Category 3a	Highly likely to be tumorigenic in rats but not in humans through prior established and well recognized mechanisms known to be human irrelevant, such that a 2-year rat study would not add value.
Category 3b	Highly likely not to be tumorigenic in both rats and humans, such that a 2-year rat study would not add value.

Each CAD had to be completed prior to or within 14–18 months of an ongoing 2-year rat study and could not be informed by any interim 2-year study data. Sponsors submitted their CADs to one of the participating DRAs (Table 2) using a dedicated email address. The submitted CADs were shared with the other DRAs. Each participating DRA independently reviewed the submitted CADs, and the rationale for concurrence or non-concurrence with the sponsor’s assessment and carcinogenicity risk category was documented. DRA review staff were blinded to the sponsor, compound identification, and the regulatory status of the pharmaceutical. In some cases, DRAs sought limited clarification regarding completeness of information from the sponsor via an unblinded assistant.

**TABLE 2:** Drug Regulatory Authority (DRA) participation in Prospective Evaluation Study.

European medicines agency (EMA)	Contributed to categorization of all 49 submitted CADs
Pharmaceuticals and medical devices agency (PMDA)	
U.S. Food and drug administration (FDA)	
Health Canada (HC)	Contributed to categorization of 41 submitted CADs after entry into PES
Swiss Agency for Therapeutic Products (SMC)	Contributed to categorization of 23 submitted CADs after entry into PES

All DRAs participated in evaluative comparison of CADs to associated 2-year rat carcinogenicity study outcomes.

Category 3a and 3b cases were considered to have the greatest potential impact on the overall outcome of the PES in terms of defining the criteria to support a WoE assessment *in lieu* of conducting a 2-year rat study as these cases would result in a conclusion that a 2-year rat study would not add value to the assessment of human carcinogenic risk. Therefore, receipt of at least 20 CADs that were categorized as either 3a or 3b from a DRA perspective (i.e., at least one DRA agreed with the sponsor’s category 3a/3b designation) was considered necessary to gain sufficient experience to support a potential revision to the ICH S1B guideline.

Initially, the DRA group included the three founding regulatory members of ICH (EMA, FDA, PMDA, September 2014) as confidentiality agreements were available between these Agencies. Periodically, DRAs met by teleconference to discuss each CAD and to assess concordance in categorizations reached by each region’s independent review of the CADs. Industry members of the EWG were not included in these discussions because of the proprietary nature of the data. However, at various timepoints the full EWG, which included Industry members, as well as DRAs that did not have mutual confidentiality agreements, convened to discuss the results (following anonymization of the data by DRAs), determine study success criteria, and to develop the framework for the ICH S1B(R1) Addendum. At the start of the PES, it was agreed to have a single agreed-upon category for each CAD (i.e., case #s 101 to 107, and 140), even in cases where unanimity for a category was not reached across the DRAs. Health Canada (HC) joined in 2015 after a confidentiality agreement was established, increasing the number of DRAs to 4. From then on, a single final category could not be based on a majority decision, since there could not always be a majority decision with 4 parties involved. Rather, the DRAs communicated any differing viewpoints with a supporting rationale to the ICH S1B(R1) EWG. This approach remained in place when Swissmedic (SMC) joined as the 5th DRA in 2016. The DRAs have reported periodically the progress of the PES in a series of Status Reports (ICH, 2016; ICH, 2017; ICH, 2019; ICH, 2021).

### Determination of rat carcinogenicity study outcome

A summary of the FSR of the completed 2-year rat carcinogenicity studies was submitted to DRAs that contained an executive summary with sufficient information to enable independent assessment of tumor outcome (e.g., tumor incidence tables and statistical analysis). When available, complete FSRs were also submitted. Outcomes of the 2-year rat studies were reviewed by DRAs without prior knowledge of the associated CAD. Each DRA evaluated the carcinogenicity study in a manner consistent with the practice in each regulatory region and concluded whether the carcinogenicity study outcome was either:

• Positive: substantive evidence of treatment-related tumors,
• Negative: no evidence of treatment-related tumors, or
• Equivocal: numerical imbalance in tumor incidence relative to concurrent control without clear relationship to treatment. For example, relation to dose-response or historical controls was unclear, statistical significance was not achieved, or a different statistical approach to tumor incidence data was applied (e.g., trend analysis vs. pair-wise testing thresholds).

Following each DRA’s independent assessment, a teleconference was held to discuss the submitted FSR summary and to seek alignment on study outcome(s). It was agreed to designate a single outcome for each FSR summary, even in cases where unanimity on study outcome was not reached across the DRAs.

### Evaluation of CAD and carcinogenicity outcome

Following assessment of tumor outcome for each 2-year rat carcinogenicity study, the associated CAD was unblinded, and the carcinogenicity study outcome was compared with the CAD’s predicted human risk category and predicted rat tumor outcome. The data for each CAD/FSR summary pair were discussed to determine if the CAD was consistent with the 2-year rat study outcome and if the 2-year rat study added value to the assessment of human carcinogenic risk. DRAs also discussed specific WoE attributes, particularly those that suggested the conduct of a 2-year rat study would add value, to determine if identified areas of uncertainty could be addressed with additional investigative studies. In March and April 2020, DRAs held a series of teleconferences to discuss each CAD/FSR summary pair in further detail and to begin to map out the WoE framework by identifying WoE attributes that would likely necessitate a 2-year rat study and those that would support a WoE assessment *in lieu* of a 2-year rat study.

## Results

### Prospective evaluation study (PES) data set

Acceptance of CADs was initiated following publication of the RND in August 2013 (ICH, 2013). A total of 49 CADs were submitted by 25 sponsors by the closing date of December 2017. In one case, interim data of an ongoing rat study was found in the CAD, and the case was subsequently excluded from the dataset. The sponsors of three CADs (two Category 2, one non-unanimous Category 3a) indicated that the associated rat carcinogenicity study report could not be submitted, leaving a total of 45 CADs with FSRs from 22 sponsors for evaluation. The 45 cases that provided complete information (CAD and associated 2-year rat FSR) comprised the final data set for evaluation in this manuscript. The data set includes 24 Category 3a or 3b CADs with associated 2-year rat FSRs, meeting one study objective of receiving at least 20 Category 3a/b cases as designated by at least one of the participating DRAs, which lead to the closing date of 31 December 2020. The investigational compounds represented approximately 18 different pharmacological targets in active development in approximately 11 different therapeutic areas or clinical indications.

### CAD categories and concordance

Table 3 summarizes the categories designated by the sponsors and the corresponding category designated by the DRAs of the 45 completed CAD/FSR summary cases. Among the 31 cases designated by the sponsor as Category 3a or 3b, at least one DRA concurred with this designation in 24 (77%) of these cases. No DRA concurred with the sponsor’s designation of Category 3a/b in 7 cases, concluding instead that the prospective WoE assessment supported the need for a 2-year rat study to adequately assess human carcinogenic risk (i.e., Category 2).

**TABLE 3:** Concordance between DRA and sponsor category designations for 45 completed CAD/FSR cases.

CAD category	Number of CADs	
	Sponsors	DRAs
1	3	3
2	11	18
3a	14	12
3b	17	12

Category 1, highly likely to be tumorigenic in humans; Category 2, tumorigenic potential for humans is uncertain; Category 3a, highly likely to be tumorigenic in rats but not in humans; Category 3b, highly likely not to be tumorigenic in both rats and humans.

As not all category designations by DRAs were unanimous, Table 4 indicates the extent of concordance among the participating DRAs in categorizing the CADs. DRAs reached a unanimous conclusion in 1 of 3 Category 1 cases and in 15 of 18 Category 2 cases. Among the 24 cases designated as Category 3a or 3b, the DRAs reached a unanimous decision in 12 cases and a non-unanimous decision, typically between Categories 2 and 3, in an additional 12 cases.

**TABLE 4:** Concordance among DRAs on category designations for 45 completed CAD/FSR cases.

CAD category	Number of CADs		
	DRAs		
	Total	Unanimous	Non-unanimous
1	3	1	2
2	18	15	3
3a	12	7	5
3b	12	5	7

### Outcome of 2-year rat carcinogenicity studies

Tumor outcomes of the 45 two-year rat carcinogenicity studies were reported as positive, negative, or equivocal. As interpreted by the DRAs, 24 studies yielded a negative outcome while 13 yielded a positive outcome in tumor incidence. An equivocal outcome was observed in 8 cases. With one exception (case #140), the sponsors of the equivocal cases reported the study outcomes as negative; thus, the sponsors designated 31 studies as negative and 14 studies as positive for tumor outcome^1^.

Table 5 represents the number of negative, equivocal, and positive tumor outcomes of the 2-year rat studies grouped by CAD category, as designated by the DRAs. The highest percentage of negative tumor outcomes was associated with Category 3b designations, consistent with this category being defined as compounds unlikely to be carcinogenic in rats or humans based on the CAD WoE evaluation. Category 3a designations were associated with a higher percentage of positive tumor outcomes, relative to Category 3b, consistent with the WoE evaluation supporting the higher likelihood of a positive tumor outcome in rats for these compounds. For Category 2 designations, where the carcinogenic potential was indeterminate based on the CAD WoE, a similar number of 2-year studies yielded a negative or positive tumor outcome.

**TABLE 5:** Tumor outcome of 2-year rat studies for cases designated as Categories 1, 2, 3a, and 3b by DRAs.

CAD category	DRA-determined 2-year rat study outcome		
	Positive	Negative	Equivocal
1	2	1^a^	0
2	6	9	3
3a	4	5	3
3b	1^b^	9	2

Category 1: highly likely to be tumorigenic in humans; Category 2: tumorigenic potential for humans is uncertain; Category 3a: highly likely to be tumorigenic in rats but not in humans; Category 3b: highly likely not to be tumorigenic in both rats and humans.

^a^ Case 123, discussed in the text below.

^b^ Case 122, discussed in the text below.

### Outcomes in relation to CAD category designation

The basis for CAD categorizations and the outcome of the associated 2-year rat carcinogenicity studies are summarized in Tables 6-12 for Categories 3b (Table 6), 3a (Tables 7, 8), 2 (Tables 9–11), and 1 (Table 12). These tables also describe the rationale underlying concordance or lack of concordance between the DRAs and sponsors, and among DRAs regarding CAD categorization and 2-year rat tumor outcomes.

**TABLE 6:** Unanimous and non-unanimous Category 3b: Comparison of WoE assessment to tumor outcome in the 2-year rat study.

Case ID	Sponsor category	DRA category	Basis for categorization	Basis for alternative category	2-year rat tumor outcome	Discussion on CAD or outcome
103	3	3b	Therapeutic Indication: MigraineTarget: G-protein-coupled receptor (novel drug target)• Literature reports no effect or potential anti-tumor effects related to drug target inhibition; negative 2-year rat data available for comparable compound• No off-target activity in secondary pharmacology screen• No histological findings of concern at clinically relevant exposures in 6- month rat study• No genotoxicity, hormonal or immunosuppressive effects	N/A	DRA: negativeSponsor: negative	The absence of drug-related tumorigenicity in the 2-year rat study supported the WoE assessment of low carcinogenic risk in rats and humans, such that a 2-year study would not add value
122	3	3b	Therapeutic indication: Cardiomyopathy and arrhythmiasTarget: Ion channel• Literature reports potential role of channel activation in promoting tumor invasiveness. Compound 122 inhibits ion channel activity• No off-target activity in secondary pharmacology screen• Increased uterine weight, abnormal contents, microscopic dilatation in 6- month rat study• No genotoxicity or immunosuppressive effects	N/A	DRA: positiveSponsor: positiveUterine carcinoma (not predicted in CAD)	Doses resulting in increased uterine weight in the 6- month toxicity study resulted in uterine carcinoma and polyps in the 2-year rat study at exposures ∼2 times the anticipated clinical exposureThe outcome of the 2-year rat study indicated that an increase in reproductive organ weights with or without histological correlates observed in a 6- month study may be evidence of functional hormonal perturbation and suggest a potential carcinogenic riskFurther investigative studies required to assess causality and human relevance for inclusion in a WoE assessment
128	3	3b	Therapeutic indication: Viral infectionTarget: Viral protein• Non-mammalian target with no mammalian equivalent• No off-target activity in secondary pharmacology screen• No histological findings of concern in 6-month rat study• No genotoxicity, hormonal or immunosuppressive effects	N/A	DRA: negativeSponsor: negative	The absence of drug-related tumorigenicity in the 2-year rat study supported the WoE assessment of low carcinogenic risk in rats and humans, such that a 2-year study would not add value
129	3	3b	Therapeutic Indication: Viral infectionTarget: Viral protein• Non-mammalian target with no mammalian equivalent• No off-target activity in secondary pharmacology screen• No histological findings of concern in 6-month rat study• No genotoxicity, hormonal or immunosuppressive effects	N/A	DRA: negativeSponsor: negative	The absence of drug-related tumorigenicity in the 2-year rat study supported the WoE assessment of low carcinogenic risk in rats and humans, such that a 2-year study would not add value
130	3	3b	Therapeutic indication: Severe asthmaTarget: G-protein-coupled receptor inhibitor (novel drug target)• Knock-out mice lacking the drug target do not exhibit findings indicative of a potential carcinogenicity risk after 1 year of observation• No interactions with receptors/transporters screen (<10 µM)• No histological findings of concern at a 54-fold human plasma exposure margin in 6-month rat study• No genotoxicity, hormonal or immunosuppressive effects	N/A	DRA: negativeSponsor: negative	The absence of drug-related tumorigenicity in the 2-year rat study supported the WoE assessment of low carcinogenic risk in rats and humans, such that a 2-year study would not add value
118	3	3b, 2	Therapeutic indication: Viral infectionTarget: Viral protein (novel drug target)Category 3b• Non-mammalian drug target with no mammalian equivalent• No off-target activity in secondary pharmacology screen• No histological findings of concern at clinically relevant exposures in 6- month rat study• No genotoxicity, hormonal, or immunosuppressive effects	Category 2• Novel drug target• Incomplete information on metabolite characterization• Inadequate assessment of off-target activity• Demonstrating a negative 2- year study outcome considered of value to risk assessment	DRA: negativeSponsor: negative	Adequacy of compound characterization for a novel target varied across DRAsFor DRAs selecting Category 3b, the absence of drug-related tumorigenicity in the 2-year rat study supported the WoE assessment of low carcinogenic risk in rats and humans, such that a 2-year study would not add valueFor DRAs selecting Category 2, the absence of drug-related tumorigenicity in the 2-year rat study resolved uncertainties identified in the WoE assessment and provided value to the assessment of human carcinogenic risk
137	3	3b, 2	Therapeutic indication: Alzheimer’s diseaseTarget: Protease (novel drug target)Category 3b• No evidence of carcinogenic concern in knock out mice lacking the drug target• No off-target activity in secondary pharmacology screen• No histological findings of concern at clinically relevant exposures in 6-month rat study• No genotoxicity, hormonal, or immunosuppressive effects	Category 2• The compound modulates a novel drug target exhibiting complex biology that has not been well characterized which precludes confident prediction of tumorigenic outcome in rats and humans• Data from knock out mice insufficient to conclude no carcinogenic concern related to drug target biology	DRA: equivocalPancreatic islet adenoma, uterine adenoma/carcinomaSponsor: negative	For DRAs selecting Category 2, the negative/equivocal outcome in the 2-year rat study characterized tumor outcome following pharmacological inhibition of the novel drug target and provided value to the assessment of human carcinogenic risk
144	3	3b, 2	Therapeutic indication: HypertensionTarget: Steroidal receptorCategory 3b• Target biology and selectivity profiles do not raise a concern• No off-target activity in secondary pharmacology screen• Histological findings of adrenal hypertrophy and/or renal juxtaglomerular cells in 6-month rat study; however, similar histological findings for compounds that target the same receptor do not show adrenal tumors in 2-year rat studies and renal tumors in 2-year rat studies were not considered human relevant• No genotoxicity, hormonal or immunosuppressive effects	Category 2• Structural dissimilarity between compound 144 and other compounds in the class limits extrapolation of 2-year rat findings to compound 144• Findings of gastrointestinal erosion/inflammation in 6- month rat study not adequately addressed	DRA: negativeSponsor: negative	Adequacy of addressing complexity of target biology sufficient for confident prediction of outcome varied across DRAsFor DRAs selecting Category 3b, the absence of drug-related tumorigenicity in the 2-year rat study supported the WoE assessment of low carcinogenic risk in rats and humans, such that a 2-year study would not add valueFor DRAs selecting Category 2, the negative outcome in the 2-year rat study resolved uncertainties identified in the WoE assessment and provided value to the assessment of human carcinogenic risk
146	3	3b, 2	Therapeutic indication: Inflammatory diseaseTarget: PhosphodiesteraseCategory 3b• Target biology and selectivity profiles do not raise a concern• No off-target activity in secondary pharmacology screen• Human metabolites adequately assessed• No histological findings of concern in 6-month dermal rat study, the intended clinical route of administration• Uterine tumors observed with similar compound in the class from oral dosing in 2-year rat study not applicable to intended dermal application (2-year dermal mouse study was negative)• No genotoxicity, hormonal, or immunosuppressive effects	Category 2• Insufficient information provided on carcinogenic potential of drug target, potential hormonal effects, and potential immunotoxic effects• Uterine granular cell tumors observed with similar compound in the class following oral dosing suggest tumorigenic potential• Potential oral exposure from dermal application in pediatric population supports conduct of rat oral study	DRA: negativeSponsor: negative	Adequacy of information provided for target biology, hormonal, and immunotoxic endpoints varied across DRAsRelevance of tumors observed following oral dosing to the route of clinical administration, and potential systemic clinical exposure, also varied across DRAsFor DRAs selecting Category 2, the negative outcome in the 2-year rat study resolved uncertainties identified in the WoE assessment and provided value to the assessment of human carcinogenic risk
136	3	3b, 2, 1	Therapeutic indication: Inflammatory diseaseTarget: Tyrosine kinase (novel drug target)Category 3b• Target biology involved in immunity, but no direct role in tumorigenesis• No pharmacologically relevant off- target activity in secondary pharmacology screen for either compound 136 or major metabolites• No histological findings of concern in 6-month rat study or in other species tested (mice, monkeys)• No genotoxic effects for parent compound or major metabolites• No hormonal effects	Category 2• Different target selectivity limits extrapolation from related congeners in class, which exhibit an inconsistent rodent tumor profile. Compound-specific assessment considered of potential valueCategory 1• Immunosuppressive profile (decreased peripheral blood lymphocyte counts, decreased lymphoid cellularity, suppression of T-cell-dependent antibody response in 6-month rat study; suppression of T-cell-dependent antibody response in 9-month non- rodent study)• Potential cross-reactivity with related kinase presents a human carcinogenicity risk that would not be further informed by a 2-year rat study	DRA: negativeSponsor: negative	Relevance of data for related congeners with differing target selectivity, some of which present a human carcinogenic risk, varied across DRAs
124	3	3b, 3a, 2	Therapeutic indication: Adjuvant cancer treatmentTarget: Tyrosine kinaseCategory 3b• Target biology related to growth inhibition and not considered a concern• No tumor findings from 2-year rat studies with congeners in class• No off-target activity in secondary pharmacology screen• Bile duct hyperplasia, a finding of concern observed in a 14-day study, was not confirmed in the 6-month study• No genotoxicity, hormonal, or immunosuppressive effects	Category 3a• Potential for hemangiosarcoma based on a numerical but non- significant increase observed in TgRasH2 mice by a human-irrelevant pathwayCategory 2• Inadequate characterization of toxicities in 6-month rat study (e.g., chronic GI inflammation, villous atrophy in the ileum, mammary gland atrophy, bile duct/liver injury)• Incomplete characterization of metabolites• Different target selectivity profile limited extrapolation of carcinogenicity data from congeners in class to compound 124• Liver, vascular tumors in 2- year rat study observed with one congener in the class was not addressed by sponsor	DRA: negativeSponsor: negative	Adequacy of information provided for relevant toxicities observed in the 6-month rat study, extent of metabolite characterization, and data for related congeners varied across DRAsFor DRAs selecting Category 3b or 3a, the absence of drug-related tumorigenicity in the 2-year rat study supported the WoE assessment of low carcinogenic risk in rats and humans (3b), or humans (3a), such that a 2-year study would not add valueFor DRAs selecting Category 2, the absence of drug-related tumorigenicity in the 2-year rat study resolved uncertainties identified in the WoE assessment and provided value to the assessment of human carcinogenic risk
149	3	3b, 3a, 2	Therapeutic indication: General absorption enhancerTarget: Fatty acidsCategory 3b• Compound related to dietary ingredient and not considered a tumorigenic risk• Gastric mucosal hypertrophy/hyperplasia in 6-month rat study interpreted as rat-specific, not being observed in dog toxicity studies• Pancreatic adenoma in 6-month rat study observed in one low dose male but not at higher doses or in a repeat 6-month study• No genotoxicity (only Ames test performed) or hormonal effects	Category 3a• Possible tumorigenicity in forestomach and pancreas through a rat-specific and human irrelevant pathwayCategory 2• Insufficient information regarding relevance and extent of dietary intake compared to exposure to compound• Gastric mucosal findings in the main 6-month rat study and occurrence of pancreatic adenoma in a supportive 6-month rat study require further characterization• Pancreatic adenoma reported in rats with related congener• Insufficient testing for genotoxicity• Inadequate plasma exposure margin assessment (rat to human)	DRA: equivocalSkin squamous adenoma/carcinoma (not predicted in CAD)Sponsor: negative	Adequacy of information regarding dietary intake and relation to compound exposure was a key point of disagreement across DRAsRelevance of gastric mucosal findings and characterization of pancreatic toxicity also varied across DRAs

**TABLE 7:** Unanimous Category 3a: Comparison of WoE assessment to tumor outcome in the 2-year rat study.

Case ID	Sponsor category	DRA category	Basis for categorization	2-year rat tumor outcome	Discussion on CAD or outcome
116	3a	3a	Therapeutic indication: Insomnia Target: neuronal G-protein-coupled receptor • Drug target is predominately expressed in brain tissue • No cause for concern based on known drug target biology and pharmacology • No evidence of a carcinogenic effect due to drug target inhibition in a 2-year rat study with a comparable compound • Antagonist binding interaction identified for 1 off-target receptor. Known pharmacology of off-target receptor not associated with tumorigenesis • Increased liver weight, hepatocellular hypertrophy, and thyroid follicular cell hypertrophy in 6-month rat study • Observed hormonal effects due to inhibition of the drug target and were not considered a cause for concern due to margins > 60-fold human exposure • No evidence of genotoxicity, or immunosuppressive effects	DRA: negative Sponsor: negative	Increased liver weight and thyroid follicular cell hypertrophy in the 6- month rat study suggested the potential for liver and thyroid tumors in the 2-year rat study due to adaptive changes related to hepatic enzyme induction that has limited human relevance. Data was provided to indicate that CYP1A2 and CYP 3a1 were induced in the 6- month study. While the predicted hepatocellular and thyroid follicular cell tumors did not occur, the absence of drug-related tumorigenicity in the 2-year rat study did not change the WoE assessment of low carcinogenic risk in humans, such that a 2-year study would not add value.
142	3a	3a	Therapeutic indication: Fungal infection Target: Sterol synthesis • No cause for concern based on known drug target biology and pharmacology • Topical application limits systemic exposure • Major human metabolites adequately assessed • Comprehensive secondary pharmacology screen not conducted. Drug class reported to affect steroid metabolism • Hepatocellular adenoma and carcinoma observed in carcinogenicity studies with other compounds in the drug class • Negative 2-year dermal mouse study and dermal rat tumor-promoter study with compound 142 were considered supportive of negligible human carcinogenic risk • Increased liver weight and hypertrophy at 86-fold human exposure and squamous cell hyperplasia in esophagus in 6-month dermal rat study • Potential for esophageal squamous cell papilloma and carcinoma resulting from observed esophageal squamous cell hyperplasia in the 6-month dermal rat study. Finding is likely due to local irritation attributed to oral ingestion of compound 142 during self-grooming and is not human relevant • Inhibition of aromatase activity in vitro, slight delay in estrus cycle in pregnant rats from subcutaneous dosing • No genotoxicity or immunosuppressive effects	DRA: negative Sponsor: negative	Liver tumors were anticipated based on 2-year rat study data with related compounds, and observed hepatocellular hypertrophy in the 6- month study at >86 times clinical exposure. The absence of drug-related tumorigenicity in the 2-year rat study did support the WoE assessment of low carcinogenic risk in humans, such that a 2-year study would not add value.
106	3a	3a	Therapeutic indication: Viral infection Target: Viral enzyme • Non-mammalian target with no mammalian equivalent • No off-target activity in secondary pharmacology screen • 2-year rat study data with drugs in class support a Category 3a designation • Negative RasH2 transgenic mouse study • Human metabolites adequately assessed • Potential for bladder tumors due to presence of crystalluria without histological change to bladder in 6-month rat study • No genotoxicity, hormonal or immunosuppressive effects	DRA: negative Sponsor: negative	The presence of needle-like crystals in urine in the 6-month rat study suggested the potential for bladder tumors in the 2- year study from a crystalluria mechanism that has limited human relevance. The absence of drug-related tumorigenicity in the 2-year rat study supported the WoE assessment of low carcinogenic risk in humans, such that a 2-year study would not add value.
109	3a	3a	Therapeutic indication: Viral infection Target: Viral enzyme • Non-mammalian target with no mammalian equivalent • Cause for concern not identified based on the outcome of rat and mouse carcinogenicity studies conducted for other compounds in the class • No off-target activity in secondary pharmacology screen • Nasal turbinate inflammation and reactive hyperplasia in the squamous mucosa of the non-glandular stomach in the 6-month rat study • No genotoxicity, hormonal or immunosuppressive effects	DRA: equivocal Granulocytic leukemia, subcutaneous fibrosarcoma (not predicted in CAD) Sponsor: negative	The presence of reactive hyperplasia in the stomach from direct drug irritation suggested the potential for squamous tumors of the stomach in the 2-year study from local irritation mechanism that has limited human relevance. The interpretation of an equivocal outcome in the 2-year rat study is based on the absence of statistical significance for both trend and pairwise tests for the numerical imbalance of granulocytic leukemia and fibrosarcoma. The observed tumor outcome did not impact the WoE assessment concluding the compound exhibits low carcinogenic risk in humans and the 2-year rat study would not add value.
117	3a	3a	Therapeutic indication: Type 2 diabetes Target: Renal co-transporter • No cause for concern based on known drug target biology • High target selectivity • No off-target activity in secondary pharmacology screen • Adrenal medullary, testicular Leydig, and renal tumors in 2-year rat studies observed with comparable compounds, via inhibition of related off-target co-transporter • Increased kidney weight and tubule hypertrophy, and increased adrenal weight and hypertrophy in 6-month rat study • No evidence of genotoxicity, hormonal or immunosuppressive effects	DRA: positive Sponsor: positive Adrenal medullary pheochromocytoma	Adrenal medullary, testicular Leydig, and renal tubule tumors were anticipated based on the reported tumor outcome in 2-year rat studies conducted with similar compounds in the class, and the observed increase in kidney weight and tubule hypertrophy, and increased adrenal weight and hypertrophy in the 6-month rat study. In the 2-year rat study adrenal tumors were noted which is consistent with the WoE assessment for this organ. Tumors were not observed in the testis or kidney. The proposed mode of tumorigenic action in rats for the drug class is mediated by inhibition of a related co-transporter, which would not occur at clinically relevant exposure to the test compound. Therefore, the outcome of the 2-year rat study did not impact the WoE assessment concluding the compound exhibits low carcinogenic risk in humans and the 2-year rat study would not add value.
135	3a	3a	Therapeutic indication: Hypertension Target: Lyase • No cause for concern based on known drug target biology • Negative tumor outcome in 2-year rat study with comparable compound • No relevant off-target activity in secondary pharmacology screen • Human metabolites adequately assessed • Crystalluria was identified in rat urine without a histopathological change to renal or bladder tissue in the 6-month rat study. Urinary crystals not detected in human samples • Liver hypertrophy without change in liver weight in the 6-month rat study • Diffuse adrenal hypertrophy ascribed to intended pharmacological activity in the 6-month rat study • No genotoxicity, hormonal or immunosuppressive effects	DRA: equivocal Adrenal medullary pheochromocytoma and Leydig cell adenoma (not predicted in CAD) Sponsor: negative	The presence of crystalluria in the 6-month rat study suggested the potential for renal/bladder tumors in the 2-year study from a mechanism that has limited human relevance. The interpretation of an equivocal outcome is based on the absence of statistical significance for the numerical imbalance of adrenal pheochromocytoma and testicular Leydig cell tumors. The outcome of the 2-year rat study did not impact the WoE assessment concluding the compound exhibits low carcinogenic risk in humans and the 2-year rat study would not add value.
139	3a	3a	Therapeutic indication: Insomnia Target: Neuronal G-protein coupled receptor • No cause for concern based on known drug target biology and pharmacology • No off-target activity in secondary pharmacology screen • Major human metabolites adequately assessed • Comparable compound with less receptor selectivity positive for liver and thyroid follicular tumors in 2-year rat study • Increased liver weight and hepatocellular hypertrophy, increased thyroid weight and follicular hypertrophy/hyperplasia in 6-month rat study • No genotoxicity, hormonal or immunosuppressive effects	DRA: positive Sponsor: positive Granulocytic leukemia, thyroid C-cell carcinoma (not predicted in CAD)	The presence of liver hypertrophy and thyroid follicular cell hypertrophy/hyperplasia in the 6-month rat study suggested the potential for liver and follicular thyroid tumors in the 2-year study based on a mechanism that has limited human relevance. In the 2-year study, liver and follicular thyroid tumors were not observed but granulocytic leukemia (males) and thyroid C-cell carcinoma (females) were observed at an exposure multiple of 66-times and 72-times, respectively, the anticipated clinical exposure. As tumors occurred at exposure margins that are not considered human relevant, the outcome of the 2-year study did not impact the WoE assessment concluding the compound exhibits low carcinogenic risk in humans and the 2-year rat study would not add value.

**TABLE 8:** Non-unanimous Category 3a: Comparison of WoE assessment to tumor outcome in the 2-year rat study.

Case ID	Sponsor category	DRA category	Basis for categorization	Basis for alternative category	2-year rat tumor outcome	Discussion on CAD or outcome
145	3a	3a, 2	Therapeutic indication: Metastatic prostate cancer Target: Steroid receptor Category 3a • Inhibition of drug target associated with reduced cell growth and increased apoptosis. Target biology involves disruption of hormonal pathway (androgen activity) leading to high sustained LHRH and LH activity • Negative RasH2 transgenic mouse study • Major metabolites adequately assessed • Histological findings in chronic rat and dog studies suggest potential for tumors in liver, thyroid, bladder, renal, testicular, adrenal, pituitary, and endometrial tissues • Tumorigenic pathway for potential liver/thyroid tumors (drug metabolism: CYP enzyme induction demonstrated) and potential renal/bladder tumors (crystalluria) considered rat-specific and human irrelevant • Tumorigenic pathways for testicular adrenal, pituitary, and endometrial tissues relate to drug pharmacology but are not relevant to intended patient population (e.g., males on LH suppressive regimens) • No genotoxicity or immunosuppressive effects	Category 2 • Mechanistic link between adrenal findings and changes in LH levels not sufficiently characterized • Compound exhibits additional mechanisms of action not observed with other compounds in the class • Inadequate information provided to link renal/bladder histological findings to drug-related crystalluria • Inadequate information regarding risk from potential functional interaction with secondary target (GABA-receptor) • Margin of exposure for hypertrophic lesions difficult to establish and may be equivalent to steady state exposure at human clinical dose	DRA: positive Sponsor: positive Leydig cell adenoma, ovarian granulosa, bladder papilloma/carcinoma, pituitary pars distallis adenoma, thymoma (not predicted in CAD), mammary fibroadenoma (not predicted in CAD)	Adequacy of information regarding target biology and relevance of histological findings of concern in 6-month rat study and of potential-off target interactions varied across DRAs. The presumption of low human carcinogenic risk was driven primarily by attributes of the indicated patient population that could not be extrapolated to a different patient population. For some DRAs, the outcome of the 2-year rat study suggested that a Category 2 designation may have been more appropriate than a Category 3a designation.
125	3a	3a, 2	Therapeutic indication: Schizophrenia Target: Multiple neuronal G-protein coupled receptors Category 3a • Tumors anticipated in mammary and pancreatic tissues of rats secondary to elevation in prolactin, considered of limited human relevance • Hepatocellular tumors anticipated based on liver hypertrophy in 6-month study, related to rat-specific drug metabolism • Human metabolites adequately generated and evaluated in non-clinical animal models • Cecal tumors anticipated based on epithelial hyperplasia in 6-month study, related to direct tissue irritation or disruption to gut microflora, considered rat-specific • No genotoxicity or immunosuppressive effects	Category 2 • Hypertrophic / proliferative lesions observed in the mammary gland of rats in the 6-month study may be attributed to a compound-related effect on prolactin secretion • Relevance of prolactin elevation to human risk of carcinogenicity is uncertain as epidemiological literature data indicates that drug- mediated prolactin enhancement may not be rat-specific and may pose a human cancer risk • Results from 2-year rat study may inform relative prolactin-related tumor risk among similar compounds in class • Inadequate characterization and relevance of cecum hyperplasia, lung phospholipidosis • Unclear rationale for expectation of pancreatic tumors in rats	DRA: equivocal Leydig adenoma (not predicted in CAD) Sponsor: negative	Adequacy of information addressing relevance of histological findings in 6- month rat study and of prolactin elevation varied across DRAs. For DRAs selecting Category 2, the outcome of the 2-year rat study resolved uncertainties related to cecum hyperplasia in the 6-month rat toxicity study. For some DRAs, while tumors were not observed in the mammary gland (hyperplasia was noted in the chronic rat toxicity study), the outcome of the 2-year rat study did not resolve uncertainties regarding human cancer risk of drug-mediated elevated prolactin levels. Considering that epidemiological data are available, there may be alternative methods to better characterize the human relevance of this finding.
131	3a	3a, 2	Therapeutic indication: Pulmonary disorder Target: Cation channel (novel drug target) Category 3a • Neither polymorphism nor gene mutation was associated with familial tumor susceptibility or with sporadic tumor development in humans or animals. Drug target null mice, drug target antisense oligonucleotides, assessment of the COSMIC database were included in the assessment of target biology • Compound selective for drug target relative to receptors in the same family • No off-target activity in secondary pharmacology screen • Human metabolites adequately assessed • Tumors anticipated in renal/bladder tissues based on crystalluria observed in the 6-month rat study. In dogs, crystalline material was observed in urine with no correlating histological changes. In humans, urinary crystals not observed at clinical drug exposures • No genotoxicity, hormonal, or immunosuppressive effects	Category 2 • Crystalluria occurs in rats and also dogs and human subjects at higher drug exposures • Literature suggests an increased risk of urinary tract cancers following renal/ureter stones • Crystalluria overlaps with site of pharmacological action (kidneys) • Value of a rat study would be establishing an exposure-response relationship and for characterizing a novel drug target	DRAs: negative Sponsor: negative	Relevance of overlap between site of crystalluria and primary site of pharmacological activity varied across DRAs. For DRAs selecting Category 3a, while tumors predicted in renal and bladder tissues were not observed, the absence of drug-related tumorigenicity in the 2-year rat study supported the WoE assessment of low carcinogenic risk in humans, such that a 2-year study would not add value. For DRAs selecting Category 2, the absence of drug-related tumorigenicity in the 2-year rat study resolved uncertainties identified in the WoE assessment and provided value to the assessment of human carcinogenic risk.
133	3a	3a, 2	Therapeutic indication: Obesity Target: Renal co-transporters Category 3a • Genome screens don’t associate target gene mutations with human cancers • Tumors anticipated in testicular and adrenal tissues secondary to changes in calcium balance in rats, through a mechanism reported to have minimal human relevance • No change in urinary calcium and calcium biomarkers observed in clinical trials, further limiting relevance of findings in rats • Negative RasH2 transgenic mouse study • Cecal hyperplasia observed in 6-month rat study related to pharmacology and is an adaptive secondary effect • No off-target activity in secondary pharmacology screen • Human metabolites adequately generated and evaluated in non-clinical animal models • No hormonal, or immunosuppressive effects • No evidence of mutagenic activity in the Ames assay, and no increase in structural chromosome aberrations in the in vitro assay in human lymphocytes • An increase in micronuclei formation observed in the in vitro and in vivo micronucleus test. Investigative studies indicated that the findings were likely due to interference with the spindle apparatus and consistent with an aneugenic mechanism • Maximum clinical exposure did not exceed exposure at 1/20th the NOEL in the rat micronucleus assay	Category 2 • Compound 133 is a mixed target inhibitor • Different target selectivity limits extrapolation of carcinogenicity data from (selective) compounds in the class to compound 133 • Incomplete characterization and assessment of intestinal (cecum) hyperplasia observed in the 6-month rat study	DRAs: negative Sponsor: negative	Relevance of data for related compounds with differing target selectivity varied across DRAs. For DRAs selecting Category 3a, while the predicted testicular and adrenal tumors did not occur, the absence of drug-related tumorigenicity in the 2- year rat study did not change the WoE assessment of low carcinogenic risk in humans, such that a 2-year study would not add value. For DRAs selecting Category 2, the absence of drug-related tumorigenicity in the 2-year rat study resolved uncertainties identified in the WoE assessment and provided value to the assessment of human carcinogenic risk.
112	3a	3a, 2	Therapeutic indication: Neurologic disorder Target: Central benzodiazepine receptor Category 3a • Target biology not associated with tumorigenic pathways, further supported by rodent tumor profile of drug class • Human metabolites adequately addressed • No off-target activity in secondary pharmacology screen • Hepatocellular and thyroid follicular tumors in rats anticipated based on increased liver/thyroid hypertrophy in 6-month and 18-month rat toxicity studies. Mechanistic studies indicated that the compound alters the pituitary-thyroid axis and increases hepatic UDPGT in rats demonstrating that the liver and thyroid findings are likely rat-specific and considered of limited human relevance • No genotoxicity or immunosuppressive effects	Category 2 • Previous (older) 2-year dietary study reported endometrial hyperplasia/polyps and alterations in mammary tissue development not seen in shorter term oral gavage studies • Added value of 2-year rat study is long-term characterization of potential hormonal perturbation	DRAs: positive Sponsor: positive Liver adenoma, thyroid follicular cell adenoma and carcinoma	Relevance of prior findings indicative of hormonal perturbation in a 2-year dietary study varied across DRAs. For DRAs selecting Category 3a, the outcome of the 2-year rat study supported the WoE of low carcinogenic risk in humans, such that a 2-year rat study would not add value. Residual uncertainty regarding hormonal perturbation was addressed from available compound-specific and drug-class specific data. For DRAs selecting Category 2, the outcome of the 2-year rat study resolved uncertainties identified in the WoE assessment and provided value to the assessment of human carcinogenic risk.

**TABLE 9:** Unanimous Category 2: Comparison of WoE assessment to tumor outcome in the 2-year rat study.

Case ID	Sponsor category	DRA category	Basis for category 2 agreement	Sponsor’s statement of expected value (from submitted CAD)	2-year rat tumor outcome
101	2	2	Therapeutic indication: Various cancersTarget: Tyrosine kinaseResidual uncertainty• Potential impact of off-target kinase inhibition on tumor risk• Duodenal tumors in rats reported with similar drug in class• Histologic changes in the duodenum in 6-month rat study. Also observed in monkeys• Histologic changes in the ovary and testes indicative of potential hormonal perturbation in 6-month rat study• Changes in hematology and clinical chemistry parameters indicative of potential liver toxicity in 6-month rat study	The 2-year rat study will likely add value to the assessment of human carcinogenic risk considering the potential for chronic treatment in the adjuvant setting, and tumors identified in a 2-year rat study with a similar drug in the class	DRA: positiveSponsor: positiveDuodenal adenocarcinoma, males and females at <0.4-fold clinical exposureNOAEL not identified
108	2	2	Therapeutic indication: Viral infectionTarget: Viral enzymeResidual uncertainty• Positive genotoxicity data (*in vivo* rat micronucleus) of uncertain human relevance• Low incidence of hemangiosarcoma in 6-month rat study• Diverse rodent tumors observed with drug class	The 2-year rat study will inform the predictive potential of the 6-month rat study for the following profile• Positive clastogenicity• No proliferative changes but observed vascular tumor• Rodent tumors in drug classThere is also value in establishing a safety margin for risk assessment and human relevance based on exposure multiples	DRA: negativeSponsor: negative
111	2	2	Therapeutic indication: Hematologic disorderTarget: Transcriptional regulatory complexResidual uncertainty• Inhibition of drug target increases transcription of pro-angiogenic and growth factors implicated in tumor progression• Lack of precedent for compounds of this drug class	The 2-year rat study will likely add value to the assessment of human carcinogenic risk based on• Absence of carcinogenicity data with other drugs in the class• Potential for tumors related to drug target pharmacology	DRA: negativeSponsor: negative
114	2	2	Therapeutic indication: obesity, type 2 diabetesTarget: G-protein coupled receptorResidual uncertainty• Human-relevant carcinogenic hazard identified from rodent genetic models and human genetic disorders• Cellular proliferation within target tissue observed in the 3-month rat study. Also observed in mice and monkeys• Unresolved hyperplasia of intestinal crypt epithelium in small and large intestines in 3-month rat study• Lack of a 6-month rat toxicology study• Lack of precedent for compounds of this drug class	The 2-year rat study will likely add value to the assessment of human carcinogenic risk by providing information on a potential carcinogenic threshold associated with pharmacologic inhibition of the drug target.	DRA: negativeSponsor: negative

119	2	2	Therapeutic indication: inflammatory diseases including psoriasisTarget: G-protein coupled receptor (novel drug target)Residual uncertainty• Unresolved renal toxicity in 6-month rat study. Also observed in mice and monkeys• Lack of precedent for compounds of this drug class	The available set of toxicological data indicates that the carcinogenic potential for humans is uncertain and the 2-year rat study will likely add value to human carcinogenic risk assessment	DRA: negativeSponsor: negative
120	2	2	Therapeutic indication: rheumatoid arthritisTarget: Lipid kinase (novel drug target)Residual uncertainty• Immunomodulatory activity with anti- and pro-tumorigenic activities• Lack of precedent for compounds of this drug class	Due to the immunosuppressive action, coupled with a lack of carcinogenicity data available for pharmaceutical compounds of this drug class, the tumorigenic potential for humans is uncertain and the 2-year rat study will likely add value to the assessment of human carcinogenic. The ability of compound 120 to increase immune surveillance may negate any tumorigenic potential arising from sustained immunosuppression	DRA: equivocalNumerical imbalance of pancreatic islet cell adenoma/carcinoma, males, at 1x clinical exposureSponsor: negative
132	2	2	Therapeutic indication: diseases with oxidative stress and pathological inflammationTarget: Serine-threonine protein kinase (novel drug target)Residual uncertainty• Carcinogenicity risk due to sustained cell survival and potential immunomodulatory activity• Tumor promotion studies in a knockout mouse model yielded mixed results• Unresolved renal, gastrointestinal, and adrenal toxicities in 6-month rat study• Lack of precedent for compounds of this drug class	The 2-year rat study will likely add value to the assessment of human carcinogenic risk based on• Absence of carcinogenicity data with other drugs in the class• Potential for tumors related to drug target pharmacology (suppressing apoptosis and/or modulation of the immune system)	DRA: positiveSponsor: positivePituitary adenoma, males and females (reduced latency, increased incidence, and lethality). NOAEL for carcinogenicity provided ∼5-fold exposure margin
138	2	2	Therapeutic indication: cholestatic disorders, non-alcoholic steatohepatitis (NASH)Target: bile acid nuclear receptor (novel drug target)Residual uncertainty• Limited information on target pharmacology• Increased liver weight in multiple species, capacity to induce CYP and bile acid transporter *in vitro*• Lack of 6-month rat toxicology study• Limited assessment on potential hormonal effects• Lack of precedent for compounds of this drug class	The 2-year rat study will likely add value to the assessment of human carcinogenic risk by identifying tumors that are potentially human relevant	DRA: positiveSponsor: positiveHepatocellular adenoma and carcinoma, hepatocholangio-cellular adenoma, males. NOAEL for carcinogenicity provided ∼9-fold exposure margin

**TABLE 10:** DRA-designated unanimous non-concordance with sponsor’s proposed Category 3a or 3b designation: Comparison of WoE assessment to tumor outcome in the 2-year rat study.

Case ID	Sponsor category	DRA category	Basis for sponsor categorization	Basis for DRA categorization	2-year rat tumor outcome	Discussion on CAD or outcome
102	3b	2	Therapeutic indication: Inflammatory diseases	Category 2	DRA: negativeSponsor: negative	The 2-year rat study was recommended by DRAs primarily due to lack of alternative proposal for assessing carcinogenicity risk of immunomodulator, and incomplete characterization of potential hormonal perturbation in female reproductive tissues
			Target: Serine-threonine protein kinaseCategory 3b• Immunomodulatory agent. Negative carcinogenicity results with another compound with the same mode of action• No histological findings of concern at in 6-month rat and 9-month monkey studies• No genotoxicity• Degenerative findings in female rat reproductive tissues interpreted as not human-relevant	• Immunomodulatory profile not sufficiently characterized to inform cancer risk• Data from similar compound could not be extrapolated due to diverse toxicity observed in class• Further immunotoxicity profiling would have given further support to the category proposed by the sponsor or may have supported Category 1 (immunosuppression)• Histological findings in female rat reproductive tissues not fully characterized		
104	3b	2	Therapeutic indication: Symptomatic amyloidosis	Category 2	DRA: negativeSponsor: negative	2-year rat study recommended by DRAs due to insufficient information on pharmacological target, metabolite profile, and genotoxicity assessment
			Target: Transport protein (novel drug target)• No evidence that target engages carcinogenicity pathways• No evidence of proliferative or hyperplastic changes in 6-month rat study• No genotoxicity, hormonal or immunosuppressive effects• Negative result in rasH2-Tg mouse study	• Novel target with an insufficiently characterized mode of action• Insufficient level of information on identification and exposure to metabolites• Uncertain genotoxicity profile based on evidence suggesting possible aneugenicity and on limitations on dose selection for the genotoxicity studies performed		
105	3b	2	Therapeutic indication: major depressive disorder	Category 2	DRA: negativeSponsor: negative	The 2-year rat study was recommended by DRAs due to insufficient information regarding relevance of toxicology findings in liver, kidneys, parotid glands, and female reproductive tissues
			Target: Ion channelCategory 3b• No evidence that target engages carcinogenicity pathways• Histological findings in 6-month rat study interpreted as human irrelevant (renal necrosis/regeneration, liver hypertrophy, parotid hyperplasia, increased ovarian weight)• Negative tumor outcome in 2-year rat studies with members of class• No evidence of genotoxicity• Bladder hypertrophy without hyperplasia observed in 26-week rat study was considered to be of no relevance to cancer risk	• Differences in selectivity and toxicity profile from drug class precludes confidence of prediction for 2-year rat study• Uncertainty or lack of mechanistic explanation underlying toxicity observed in liver, kidneys, and parotid glands• Effects on ovaries and inhibition of prolactin also limit possibility of agreeing on a Category 3		
140	3b	2	Therapeutic indication: Neuropathic pain	Category 2	DRA: equivocal	The 2-year rat study was recommended by DRAs due to unresolved human relevance of tumors reported for some compounds in the same drug class
			Target: Ion channelCategory 3b• Target pharmacology similar to known class profile• Erosion and/or ulceration of the forestomach and glandular stomach and thickening of the forestomach mucosa were considered to be caused by chronic irritation and should not be considered relevant for human risk• Hypertrophy of hepatocytes with possible induction of liver enzyme induction were considered rat specific• No genotoxicity• Persistent estrus observed in a developmental and reproductive study was considered to be of no relevance to hormonal perturbation• No evidence of immunosuppressive effect• No histopathological changes of concern in 9-month monkey study	• Tumor profile of class is mixed, includes occurrence of pancreatic acinar cell carcinoma in male rats of uncertain human relevance• The mechanisms by which the tumors are induced are unclear and cannot be the basis for considering the tumors irrelevant to humans• Literature points to carcinogenic potential for a drug of the same class	Sponsor: positiveUrinary bladder papilloma	
148	3b	2	Therapeutic indication: Cancer	Category 2	DRA: negative	The 2-year rat study was recommended by DRAs due to uncertainty of rat tumor outcome based on complexity of drug pharmacology, and on insufficient information addressing relevance of hormonal perturbation identified in female rats
			Target: Tyrosine kinaseCategory 3b• Intended and off-target activities not linked to pro-tumorigenic pathways but may be anti-tumorigenic• No genotoxicity• No evidence of immunosuppressive effect• No histopathological changes of concern in 9-month dog study• Estrus/fertility findings in female rats not considered relevant to tumor risk• Negative result in rasH2-Tg mouse study	• Multiplicity of drug targets precludes confident prediction of tumor outcome in rats• Extrapolation of findings from class not warranted based on differences in pharmacology• Potential impact of hormonal changes detected in the rats (LH/FSH) were not sufficiently addressed• In the 26-week rat study, increases in hemorrhagic cystic degeneration in the lymph nodes which might be related to hemangiosarcomas in female rats	Sponsor: negative	
107	3a	2	Therapeutic indication: Viral infection	Category 2	DRA: positiveSponsor: positive	The 2-year rat study was recommended by DRAs due to insufficient information regarding relevance of gastrointestinal proliferative findings, metabolite profile, and uncertainty regarding product specificity
			Target: Viral polymeraseCategory 3a• Expectation of liver tumors in 2-year rat study based on increased liver weight/hypertrophy in 26-week rat study via a rat-specific mechanism• Gastrointestinal epithelial proliferation observed in 6-month rat study at high multiple of clinical exposure• No evidence of genotoxicity, hormonal perturbation, and immunosuppressive effects• No histopathological changes of concern in 39-week dog study	• Hyperplastic gastrointestinal findings not sufficiently characterized to address time-dependence of exposure/response or to allow consideration of the dog study where such toxicity was not observed• Lack of precedent for compounds of this drug class• Off-target activity identified in secondary screen raised concern of product specificity• Limited information was provided on metabolites	Liver adenoma	
141	3a	2	Data not disclosed	N/A	DRA: positive	Positive tumor response was partially consistent with Sponsor’s expectation of outcome
					Sponsor: positive	

**TABLE 11:** DRA-designated non-unanimous Category 2: Comparison of WoE assessment to tumor outcome of the 2-year rat study.

Case ID	Sponsor category	DRA category	Basis for categorization	Basis for alternative category by DRA(s)	2-year rat tumor outcome
126	2	3a, 2	Therapeutic indication: major depressive disorder	Category 3a	DRA: negative
			Target: Neuronal ion channelCategory 2• Non-selective nature of the compound, raising uncertainties in extrapolating carcinogenicity outcomes from others in the drug class• Evidence of hormonal disruption (literature data)• Potential immunosuppressing effects• Uncertainties in the characterization of *N*-nitroso metabolite• Exposure reached in the repeated-dose toxicity studies were not in excess of clinical exposure levels	• Lack of proliferative findings in the systemic toxicity studies• Hormonal effects were not seen in the submission data and this overrules literature• Local exposure resulting in nasal cavity findings in animals are not reached in humans• Carcinogenicity study in rat is unlikely to add value on the definition of human risk for the above-mentioned local effect and immune suppression-related carcinogenicity	Sponsor: negative
127	2	3a, 2	Therapeutic indication: Alzheimer’s disease	Category 3a	DRA: equivocal
			Target: B-amyloid protein (novel drug target)Category 2• Not a well understood novel target• Potential for carcinogenic risk in rat liver and possibly other organs	• On-target pharmacology not linked in principle to anti pro-tumorigenic pathways• No genotoxicity, hormonal perturbation or immunosuppression• No evidence indicating a potential for neoplasia in the rat chronic toxicity study• Non-rodent studies in Cynomolgus monkeys did not show any toxicological findings indicating a potential for neoplastic events	Increased testicular Leydig adenoma and hepatocellular adenomaSponsor: negative
121	2	2, 1	Therapeutic indication: Hematologic disorderTarget: Tyrosine kinase and Serine-threonine kinase receptorCategory 2• Immunosuppressive activity• Uncertainties regarding action on specific target and the lack of experience regarding molecules acting on this receptor• Incomplete characterization of the major human metabolite• Heterogeneity in toxicology profile of this class of drugs limits possibility of extrapolation	Category 1• Pharmacodynamic effects of the drug (i.e., immunosuppression)• Rodent bioassays have shown limited value in defining this type of carcinogenic risk• Malignancies observed with compounds of the same class (i.e., tofacitinib)• The lack of other risk factors such as genotoxicity, increases in neoplasia in 6-month transgenic mouse study• Difficulties in metabolite characterization, as exposure levels low in rodents	DRA: positiveSponsor: positiveTesticular Leydig adenoma

**TABLE 12:** Unanimous and non-unanimous Category 1: Comparison of WoE assessment to tumor outcome in the 2-year rat study.

Case ID	Sponsor category	DRA category	Basis for category 1	Basis for alternative category by DRA(s)	2-year rat tumor outcome	Discussion on CAD or outcome
113	1	1, 2	Therapeutic indication: Various types of cancerTarget: Transcriptional regulatory proteinCategory 1• 6-month study shows pilomatricoma• Complex pharmacology underlying Shh and catenin signaling, proposed mode of action resulting in pilomatricoma• No genotoxicity, no immunosuppressive effects	Category 2• Complex pharmacology underlying Shh and catenin signaling and the proposed mode of action resulting in pilomatricoma raised concerns of over- or under-estimating human risk• Potential off-target effects• Potential hormonal effects (increase in FSH and LH)• Discussion on safety margins not sufficient• Experience with drug class insufficient to aid prediction for the compound	DRA: positiveSponsor: positivePilomatricoma, keratoacanthoma	The 2-year study outcome was consistent with the sponsor’s prediction of mechanism-based pilomatricomaResults of the study addressed the DRA’s concern of over- or under-estimating human risk from a mechanism-based prediction, and therefore added value to the overall assessment of human carcinogenicity risk
123	1	1, 2	Therapeutic indication: Rheumatoid arthritisTarget: Tyrosine kinaseCategory 1• Genotoxicity findings indicate a potency to induce polyploidy• Immunosuppression in the repeated-dose toxicity study in rats• Malignancies observed with compounds of the same class (tofacitinib)• In monkeys tofacitinib induced lymphoma, related to immunosuppressive effect• Tumors reported in patients treated with JAK1/2 inhibitors, tofacitinib and ruxolitinib	Category 2• Proliferative findings in stomach and renal tubules were considered preneoplastic changes• Hibernoma observed with tofacitinib• Address the potential for ‘off target’ tumors, despite the recognized malignancy risk from immunomodulation, for which rodent studies are considered poorly predictive• The effect of compound on prolactin signaling, as observed with the class, was not evaluated• Exposure associated with polyploidy at expected human therapeutic exposure unclear• Human cancer data described with tofacitinib considered not robust	DRA: negativeSponsor: negative	In this case, the 2-year rat study and transgenic mouse study are considered poor predictors for carcinogenic risk in humans due to immunosuppressionBecause tumors have been reported in patients treated with pharmaceutical class, the compound may exhibit tumorigenic effects in humans and could be labeled accordingly
143	1	1	Data not disclosed	N/A	DRA: positive	N/A
					Sponsor: positive	

**TABLE 13:** WoE attributes associated with DRA-designated unanimous Category 3a and 3b cases.

WoE factor	Attribute supportive of category 3a or 3b designation
Target Biology	Target biology is well characterized and not associated with cellular pathways known to be involved with human cancer development. Often, the pharmaceutical target was non- mammalian and carcinogenicity data were available with the pharmacologic drug class
Secondary pharmacology	No identified concerns from secondary pharmacology screens intended to inform off-target potential for the pharmaceutical
Histopathology data from chronic studies	Results from 6-month rat chronic toxicity studies indicate no hyperplasia, hypertrophy, atypical cellular alterations, or degenerative/regenerative changes without adequate explanation of pathogenesis or human relevance, indicative of no on- or off-target potential of carcinogenic concern
Hormonal effects	No perturbation of endocrine and reproductive organs observed, or endocrine findings adequately explained with respect to potential human relevance
Genotoxicity	The overall assessment of genotoxic potential is concluded to be negative
Immune modulation	No evidence of immune modulation or immunotoxicity based on target biology and repeat- dose toxicology studies in rats

#### Category 3b

Category 3b was designated when the prospective WoE assessment supported a conclusion that the predicted carcinogenic risk is low or absent for both rats and humans, such that the outcome of a 2-year rat study would not add value to the assessment. The sponsors designated 17 cases as Category 3b of which the DRAs agreed fully or partially with 12 of those cases (Tables 3, 4). Among these 12 Category 3b cases, 11 were reported by the sponsors as having a negative tumor outcome, and 1 case was reported as positive (Table 6). The DRAs assessed two sponsor-designated negative cases as equivocal. In one equivocal case (#137), there was a dose-dependent numerical imbalance in the incidence of pancreatic islet neoplasms which exceeded the historical range in the high-dose group but was not statistically significant by trend or pairwise testing. In addition, a dose-independent numerical imbalance for uterine endometrial neoplasms showed an incidence higher than in the concurrent control group but remained within the historical rate for the test site. In the second equivocal case (#149), there were numerical imbalances in 3 dermal neoplasms (squamous cell papilloma, carcinoma, and keratoacanthoma) in males that reached statistical significance only when combined, driven primarily by a higher incidence of keratoacanthoma at the high dose. The latter exceeded the historical control for rats from the study site.

The tumor outcome of one Category 3b case was determined to be positive and treatment-related by both the sponsor and the DRAs (case #122) for uterine carcinoma. Retrospective examination of the 6-month toxicology study revealed a marked increase in uterine weight with abnormal contents, with microscopic evidence of dilatation at doses that were associated with uterine neoplasms in the 2-year study. The occurrence of uterine carcinoma at low multiples of clinical exposure in the 2-year rat study was not consistent with the original WoE assessment of low carcinogenic risk (Category 3b). At the time of CAD assessment, these uterine findings in the 6-month study were not recognized as a risk factor for development of uterine neoplasia by the sponsor or DRAs. It is now noted that an increase in reproductive organ weights with or without histological correlates observed in a 6-month study may be interpreted as a predisposing risk factor for neoplasia upon long-term administration. Further investigative studies to understand the underlying mechanism and human relevance would be appropriate in such cases as part of a WoE evaluation in determining whether a 2-year rat study is warranted.

#### Category 3a

Category 3a was designated when the prospective WoE supported a conclusion that the predicted cancer risk is low in humans, but that a positive tumor outcome is likely in the 2-year rat study by a species-specific and human irrelevant pathway. The sponsors designated 14 cases as Category 3a of which the DRAs agreed either fully or partially with 12 of those cases (Tables 3, 4). Among the 12 cases designated as Category 3a by the DRAs, 4 yielded a positive tumor outcome in the 2-year rat study as assessed by the DRAs and sponsors. Another 5 cases yielded a negative tumor outcome as assessed by the sponsors and DRAs, and 3 cases yielded an equivocal tumor outcome as assessed by the DRAs. The sponsors of the 3 DRA-designated equivocal cases interpreted the 2-year rat studies as being negative (case #s 109, 135 of Table 7; case 125 of Table 8). In some cases, tumor types that were observed in the 2-year rat study were not anticipated based on the WoE assessment (case #s 109, 125, 135, 139, 145), and not all tumor types anticipated from the WoE assessment were observed in the 2-year rat study (case #s 106, 109, 116, 117, 125, 131, 133, 135, 139, 142, 145 of Tables 7 and 8). However, none of the tumor types observed in the positive studies were interpreted as presenting a human carcinogenicity risk due to either human irrelevance based on anticipated tumorigenic mechanism and/or the high exposure multiple at which tumors emerged.

#### Category 2

Category 2 was designated when the prospective WoE assessment indicated that human carcinogenic risk is uncertain, and results from a 2-year rat study would add value to the assessment. Sponsors submitted 11 CADs with a Category 2 designation and the DRAs unanimously agreed with the sponsor’s designation in 8 of those cases. Table 9 lists key observations recognized by both sponsors and the DRAs as presenting substantial uncertainty regarding human carcinogenic risk, and describes the anticipated value of the 2-year rat study to the overall risk assessment. In each case, uncertainty was identified from more than one WoE factor and often derived from several observations. In general, substantial uncertainty was identified from the compound’s pharmacological mechanism or compound-specific toxicology findings and the absence of information from rat carcinogenicity studies with other compounds of the drug class. In one case (#108), a diverse rodent tumor profile associated with the drug class contributed to the concerns identified from compound-specific findings of potential genotoxicity and a low incidence of vascular tumors in the chronic rat toxicology study. In another case (#114), a 3-month rat study was submitted as the longest repeat-dose toxicity study in the WoE assessment, and no data were submitted following 6 months of repeat-dosing in rats. Given these uncertainties, a positive or negative tumor outcome in the 2-year rat study would be interpreted as adding value to the overall assessment of human carcinogenic risk.

For the 8 cases unanimously designated as Category 2 by the DRAs and sponsors, a positive tumor outcome, as interpreted by both the DRAs and the sponsor, was observed in 3 of the 8 cases. These tumor outcomes consisted of duodenal adenocarcinoma (case #101), hepatocellular and hepatocholangiocellular adenoma (case #138), and pituitary adenoma (case #132) (Table 9). The sponsor reported a negative tumor outcome for the remaining 5 cases and the DRAs agreed with this interpretation in 4 cases, citing an equivocal outcome for 1 case (#120) based on a numerical imbalance of pancreatic islet adenoma and carcinoma.

For 7 cases submitted by sponsors proposing a Category 3a or 3b designation, the DRAs placed these cases unanimously in Category 2 because of identified concerns not sufficiently addressed in the CAD. A 2-year rat study would be warranted to establish an adequate assessment of carcinogenic risk in these cases (Table 10). In many of these cases, DRAs cited insufficient information regarding the relevance of histological findings identified in the 6-month rat study to potential human carcinogenic risk (e.g., hypertrophy, hyperplasia, injury/regeneration of various tissues). Findings indicative of hormonal perturbation in rats without sufficient explanation was additionally cited in three cases (#s 102, 105, 148). Additional reasons included insufficient knowledge of drug target pathways given the novelty of the target or the multiplicity of drug targets, and insufficient information provided on metabolite profiles, genetic toxicology testing, and uncertain relevance of experience with the associated drug class. For one case (#102), the CAD did not include sufficient information about the compound’s immunomodulatory activity or an adequate characterization of a signal in female reproductive tissues for the DRAs to concur with the sponsor’s conclusion of low human risk and category 3b designation.

Among these 7 cases, a negative tumor outcome in the 2-year rat study was observed for #s 102, 104, 105, and 148, and a positive or equivocal tumor outcome was observed for #s 140, 107, and 141. For case #140, a potential signal of urinary bladder papilloma was reported in the 2-year study which was not anticipated in the CAD despite the occurrence of bladder hypertrophy in the 6-month toxicology study. Hepatocellular adenoma was observed in the 2-year rat study for case #107 and was consistent with the sponsor’s expectation of liver tumors based on increased liver weight/hypertrophy in the 6-month toxicology study. Details of case #141 are undisclosable; however, the positive tumor outcome was only partially consistent with the sponsor’s expectation in the CAD.

For three cases where the sponsor submitted a Category 2 designation, the DRAs did not reach unanimous alignment, with one or more DRAs concluding that a 2-year rat study would not add value to the WoE assessment (Table 11). In one case (#115), the DRAs did not align on the relevance of compound-specific findings indicative of hormonal disruption and potential immunosuppression, or whether a 2-year rat study would provide adequate resolution to those concerns. The tumor outcome in this case was negative. In another case (#127), the DRAs differed on whether sufficient knowledge was available for the drug target to allow an adequate assessment of a pharmacology-based carcinogenic risk. The sponsor interpreted the 2-year rat study as being negative, whereas the DRAs interpreted the study as potentially positive for Leydig cell tumors and liver adenoma. In the final case (#121), the DRAs did not align on whether results from a 2-year rat study would adequately address the concern of immunomodulation related to the compound’s pharmacological mechanism. The 2-year rat study outcome in this case was positive for Leydig cell adenoma.

#### Category 1

Category 1 was designated when the prospective WoE assessment supported the conclusion that the predicted carcinogenicity risk is highly likely in humans such that a product would be labeled accordingly and a 2-year rat, mouse, or transgenic mouse carcinogenicity study would not add value. The sponsors submitted 3 CADs with a Category 1 designation (Table 12), and the DRAs unanimously agreed with the sponsor’s designation in only 1 of those cases (#143, data not disclosable). In all cases, carcinogenic potential was predicted from human carcinogenicity data available from the drug class. In two cases (#s 113, 123), some DRAs concluded that the conduct of a 2-year rat study would be appropriate, based on inadequate information provided for several WoE factors and a presumption that additional data would further inform the extent of human carcinogenic risk.

The carcinogenicity study outcome of case #113 was considered positive by both the sponsor and DRAs, which was consistent with the sponsor’s prediction of pilomatricoma, and with an additional observation of keratoacanthoma. For another case (#123), the rat carcinogenicity study was negative which, for some DRAs, de-risked observed proliferative findings in the stomach and renal tubules that were not considered related to potential immunosuppressive effects. The third case (#143) yielded a positive tumor outcome as determined by the sponsor and DRAs; however, additional details of this case are not disclosable.

## Discussion

If a new pharmaceutical will be used as continuous therapy for 6 months or longer, or if the drug will be used intermittently for a duration of time that represents a minimum of 6 months in total, evaluation of human carcinogenic risk is recommended before licensing a marketing authorization in most cases (ICH, 1995). To this end, ICH S1B recommended that the carcinogenic potential of a pharmaceutical be evaluated in in vivo 2-year carcinogenicity studies with rats and mice. Alternatively, the 2-year mouse study can be substituted with an *in vivo* six-month study with transgenic mice. This testing strategy has been common practice since adoption of ICH S1B in 1997 and, with some exceptions, was applied to investigational pharmaceuticals regardless of drug target, compound-specific toxicology, or prior human or animal carcinogenicity data available for the drug class. Given the evolutions in understanding of potential mechanisms leading to the development of neoplasms (Hanahan and Weinberg, 2011) and the recognized limitations inherent to rodent carcinogenicity studies, during the last decades, several publications have discussed the need for refinement or alternatives to the conduct of one or both *in vivo* carcinogenicity studies (Bourcier et al., 2015; Cohen, 2004; Goodman, 2001; Reddy et al., 2010; Sistare et al., 2011; Van der Laan et al., 2017; Woutersen et al., 2016; Cohen et al., 2019).

### Process-related remarks

The RND that initiated the PES included a description of WoE factors that should be addressed in a CAD (ICH, 2013). These recommendations were informed by prior retrospective studies that identified pharmacological and toxicological attributes of pharmaceuticals that correlated with a negative or positive tumor outcome in 2-year rat studies (Sistare et al., 2011; Van der Laan et al., 2016a; Van der Laan et al., 2016b). The PhRMA dataset (Sistare et al., 2011), which formed the primary basis for the prospective evaluation study, consisted of 182 compounds, and an additional 76 compounds were later included from the IARC dataset. The PhRMA dataset (without IARC data) was enlarged by data from FDA and JPMA to approximately 255 compounds (Van der Laan et al., 2016a). Another dataset of 289 compounds was analyzed later that year (Van der Laan et al., 2016b). In the study presented herein, these attributes were applied in a prospective manner to predict the outcome and potential value of 2-year rat studies that had not yet been completed. This was achieved by explicitly directing sponsors to submit CADs only for programs where the 2-year rat studies had not progressed beyond 18 months of dosing, and without including any interim information that might be available from the ongoing 2-year study. To further minimize bias, the acceptable in-life phase was reduced from 18 to 14-month for all CADs effective 1 June 2016. Sponsors were to include the date of initiation of the 2-year rat study and the date of completion of the CAD. Most (60%) CADs were prepared during months 13–18 of the 2-year rat study, while 40% were prepared during the first 12 months of dosing.

The quality of the submitted CADs was variable. In some cases, the CAD addressed all weight of evidence factors outlined in the RND with sufficient detail to enable a well-informed assessment of the potential outcome and value of the 2-year rat carcinogenicity study. In other cases, information was either insufficient or missing from the CAD. Some examples of deficiencies include:

• insufficient description of the pharmacological target, downstream pharmacological effects, and drug target biology,
• incomplete description of receptor targets in secondary pharmacology studies,
• inadequate assessment of histological findings of concern,
• margins of exposure were not discussed,
• insufficient information regarding mechanism for cited rodent-specific effects,
• lack of detail regarding metabolism of parent compound and properties of metabolites, including identification of human metabolites, and
• insufficient, incomplete, or no discussion of other compounds in the drug class.

In three cases, additional information was requested from the sponsor as the CAD lacked data to an extent that it precluded a sufficient assessment of potential outcome and value of the 2-year rat carcinogenicity study.

The 45 CADs that comprise the final dataset were self-selected by the participating sponsors. The RND called for submission of CADs for ‘all investigational pharmaceuticals subject to 2-year rat carcinogenicity studies under current ICH S1A Guideline’ but also emphasized that submission of CADs designated as Category 3a and 3b would be of key importance, as these cases represent the most notable departure from current carcinogenicity testing guidelines. Therefore, the PES dataset may be biased toward investigational drugs where sponsors concluded that a 2-year rat study is not warranted for assessing human carcinogenic risk. Whether these cases are representative of all investigational drugs requiring a carcinogenicity risk assessment is unknown, yet consideration of the WoE factors can be reasonably applied to all such investigational drugs. Based on the number of cases where DRAs unanimously agreed with the sponsor’s designation of a CAD as 3a, or 3b in the PES dataset, approximately 27% of 2-year rat studies could have been avoided by applying the WoE approach (12 unanimous Category 3a/3b divided by 45 CADs submitted).

### Category 3b and 3a

The framework recommended in the S1B(R1) Addendum (ICH, 2022) was principally supported from evaluation of Category 3a and 3b cases in the PES. These carcinogenicity risk categories postulated that data from a 2-year rat study would not add value because the WoE assessment is sufficiently persuasive to conclude that human carcinogenicity risk is unlikely.

The presumption for Category 3b was that a 2-year rat study would yield a negative tumor outcome and therefore not contribute further to the conclusion of minimal human risk based on the WoE assessment. The negative or equivocal tumor outcomes seen for 11 of the 12 DRA-designated Category 3b cases are consistent with this presumption. Similar results were observed with the 17 Category 3b cases as designated by sponsors, wherein 15 yielded a negative tumor outcome. A review of the Category 3b cases, with a particular focus on the unanimous cases, identified common attributes that aligned with a negative 2-year rat study and are summarized in Table 13. These attributes included 1) a target biology that is well-characterized and not known to be associated with carcinogenic pathways. Often, the availability of carcinogenicity data in rats from other class members supplemented the conclusion that an investigational drug’s target biology would not be of carcinogenic concern; 2) High target selectivity as assessed by sufficiently broad secondary pharmacology screens. Such screens would preferentially include targets of higher *a priori* concern, such as hormone receptors and targets with known carcinogenic liability; 3) an absence of histological changes in chronic (6-month) rat toxicology studies indicative of carcinogenic concern, notably hyperplasia, hypertrophy, atypical cellular alterations, and degenerative/regenerative findings. If such findings are present, they are demonstrated to be human irrelevant; 4) an absence of perturbation to endocrine and reproductive organs, including changes to reproductive organ weights; 5) a negative battery of genotoxicity studies based on criteria from the ICH S2 (R1) guideline (International Council for Harmonisation, 2011), and 6) no evidence of immune modulation or immunotoxicity.

As noted above, the occurrence of a negative tumor outcome for Category 3b cases was similar whether the category was designated by the sponsor or by the DRAs. However, DRAs were more likely than sponsors to designate a compound as Category 2, suggesting that DRAs were more conservative than sponsors in accepting the WoE without 2-year rat data in some cases (Table 10). A more conservative position than proposed by the sponsor was driven by at least 2 DRAs, with one exception where a single DRA took a more conservative position than the other DRAs (#112). The identity of >2 DRAs generally varied across cases. A more conservative approach was also partly due to the limited ability of both DRAs and sponsors to fully investigate signals of concern identified in the WoE assessment within the confines of the PES. For example, as seen in cases #s 102, 104, 141, and 107 of Table 10, the sponsor’s WoE assessment did not provide adequate information for several WoE factors, such as target biology, general toxicity, and genetic toxicity, which could not be readily addressed by the sponsor during the PES. However, a sponsor would have greater latitude in a ‘real-world’ situation to clarify and supplement the WoE assessment, as needed, to address deficiencies identified by the reviewing DRA. In other cases, the issues cited by the DRAs were more substantial and difficult to resolve, and also reflect a more conservative risk tolerance relative to the sponsor (e.g., case #s 105, 140, and 148 of Table 10). For example, in one case (#140), the DRAs cited the unresolved human relevance of a known positive tumor profile for a drug class as not being consistent with a Category 3b designation, and for another case (#148) the complexity of drug pharmacology precluded confident prediction of the 2-year rat tumor outcome and value, necessitating the conduct of a 2-year rat study. Of note, the tumor outcome of these cases, both negative and positive, can be reasonably viewed as adding value to the overall WoE assessment of human risk.

Unlike Category 3b, the presumption for Category 3a was that the 2-year rat study would likely result in a positive tumor outcome through a prior established and well-recognized mechanism considered to be human irrelevant. A positive tumor outcome by a human-irrelevant pathway would therefore not contribute further to the conclusion of minimal human risk based on the WoE. The prediction of a positive, human-irrelevant tumor outcome for the 7 unanimous DRA-designated Category 3a cases was most frequently based on histological findings indicative of a hyperplastic and/or a hypertrophic response in the 6-month rat toxicology study (e.g., increased liver weight/cellular hypertrophy in cases #116, 142, and 139). In 2 cases (#106, 135), the expectation of bladder tumors was based on the presence of urinary crystals without histological changes to the urothelium. Available information on the tumor outcome for drugs with a similar pharmacological mechanism also contributed to the positive prediction in some cases (e.g., #142, 117).

The actual tumor outcome from the 2-year rat studies for these compounds indicates that predicting a positive tumor outcome with organ specificity based on 6-month toxicology data remains a challenging proposition, consistent with prior reports (Jacobs, 2005; Sistare et al., 2011). It should be noted that the absence of an anticipated tumor type from a 2-year rat study is not interpreted as being a contrary outcome, as one is predicting the probability and not the certainty of tumor emergence in a given organ. Of more concern are cases where tumor types emerged that were not anticipated from the WoE analysis in the CAD. For example, the occurrence of granulocytic leukemia and thyroid C-cell adenoma for case #139 clearly differs from the anticipated tumor types of liver and thyroid follicular tumors based on histological changes to these organs in the 6-month toxicology study. The unanticipated tumors emerged at exposure multiples of 66-times and 72-times clinical exposure, respectively, and therefore did not change the overall assessment of low human carcinogenic risk based on the prospective WoE. The tumor outcome of 2 additional unanimous Category 3a cases (#s 109, 135) was also discordant from the tumor types anticipated based on the WoE. However, the tumor signal in these cases was not persuasive, and the studies were interpreted as negative by the sponsors. While the outcome was interpreted as ‘equivocal’ by the DRAs, there was also agreement that the equivocal outcome did not change the overall assessment that human carcinogenic risk was unlikely based on the prospective WoE. That these unanticipated tumor types did not change the assessment of human carcinogenic risk is reassuring of safety for applying this WoE approach to drug candidates with similar pharmacological and toxicologic profiles. However, these cases demonstrate that positive prediction is less reliable than negative prediction of tumor outcome and, as such, may merit a more conservative evaluation of the WoE regarding the necessity of a 2-year rat study.

### Category 2

**FIGURE 2:**
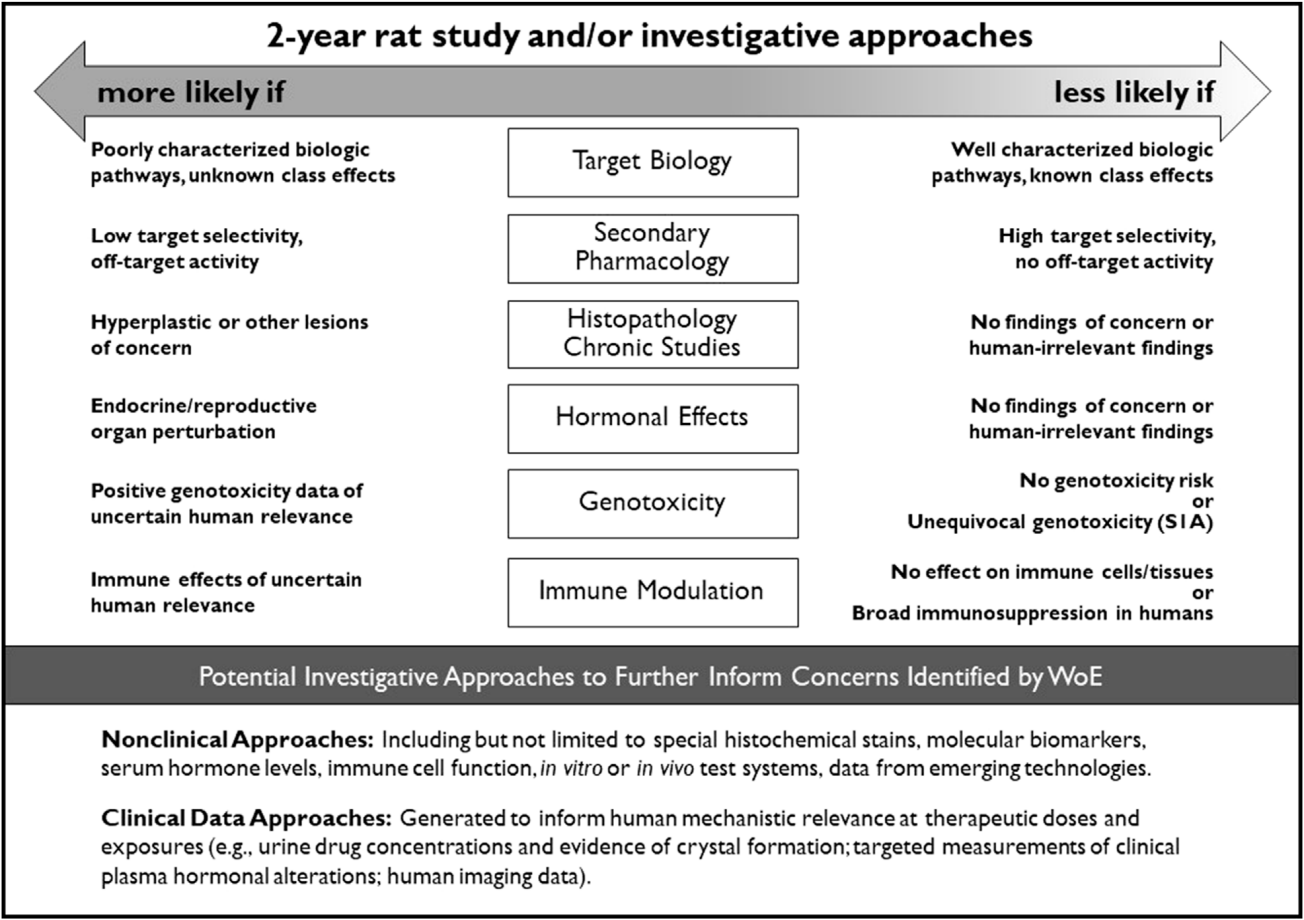
Integration of key WoE factors and potential investigative approaches to further inform on the value of conducting a 2-year rat study for assessment of human carcinogenic risk.

Sponsors and DRAs unanimously agreed in 8 cases that the conduct of a 2-year rat study would be appropriate to address uncertainties identified in the CAD (Table 9). These unanimous decisions aided in defining common WoE attributes that introduced significant uncertainty into predicting the outcome and/or value of a 2-year rat study. These attributes are generally captured in Figure 2 of the ICH S1B(R1) Addendum which provides guidance on integration of the key WoE factors.

For most cases, the sponsors cited drug target pharmacology and the known tumor profile from other class members as a cause for concern which merits the conduct of a 2-year rat study, rather than compound-specific toxicology findings. As captured by the sponsor’s statements in the CAD, a 2-year rat study was anticipated to establish a threshold of tumorigenic activity, if present, and to identify an exposure margin that would allow an exposure-based assessment of human relevance and carcinogenic risk on a compound-specific basis. Among the 4 cases with a positive outcome as determined by the DRAs, two yielded carcinogenic exposures that were lower than clinical exposure, and two identified non-carcinogenic exposures that were 5-fold and 9-fold higher than clinical exposure. The absence of a safety margin for the former two provided the sponsor with further evidence of potential risk in addition to concerns identified with the drug class (case #101) and with the pharmacological mechanism (case #120). The presence of a safety margin for the latter two provided the sponsor with empirical evidence that mitigated carcinogenic risk raised by concerns identified in the CAD (case #s 132, 138). For other cases, studies that yielded a negative tumor outcome provided the sponsor with evidence of safety that would be integrated with other data in the overall WoE evaluation of human risk.

In their analyses of the unanimous Category 2 cases, in addition to drug target-based concerns, in some cases the DRAs cited literature reporting both pro- and anti-tumor activities of the drug target which precluded both confident prediction of human risk and rat tumor outcome. The DRAs also frequently cited more compound-specific toxicology findings with inadequate explanations of causality and human relevance as additional reasons to conduct a 2-year rat study. In practice, further investigative approaches may be applied to address the human relevance of concerns identified in the WoE assessment and, if adequately de-risked, may negate the value of conducting a 2-year rat study. The feasibility of this approach would depend on the type and number of concerns identified in the WoE assessment; for example, concerns identified for several WoE factors would be more challenging to de-risk with investigative approaches compared to a concern identified for a single WoE factor. A multiplicity of concerns was generally identified for the unanimous Category 2 cases. For such cases, the DRAs noted that a negative tumor outcome or identification of a carcinogenic threshold in a 2-year rat study can add particular value to the overall assessment of human risk.

### Category 1

The current ICH S1A guideline (ICH, 1995) recommends that long term carcinogenicity studies are not needed to inform human cancer risk from compounds that exhibit unequivocal genotoxic activity. The S1A guidance, however, does not address non-genotoxic carcinogenic mechanisms that are recognized or presumed to have human relevance (Al-Zoughool et al., 2019; Krewski et al., 2019). Principal among these non-genotoxic mechanisms includes compounds that are broadly immunosuppressive, result in persistent hormonal perturbation, or otherwise engage cell growth/survival pathways that lead to persistent cell replication.

The PES dataset includes three compounds submitted as Category 1 by sponsors based on arguments related to immunosuppression for two cases and a persistent rebound proliferative response for one case. The DRAs unanimously agreed to this categorization for one case based on persuasive evidence of broad immunosuppression. For the remaining two cases, some DRAs concluded that data from a 2-year rat study would provide additional value while also acknowledging the likely human risk based on the pharmacological mechanism of each compound. In one case (#113), some DRAs were concerned that the sponsor’s prediction of a benign tumor type underestimated the risk of inducing more serious malignancies, a potential outcome that could be addressed in a 2-year rat study. The tumor outcome was restricted to only benign tumor types which mitigated the concern for other malignancies and was considered an outcome of value by some DRAs. In another case (#123), some DRAs cited concerns of potential tumorigenesis arising from mechanisms unrelated to the compound’s immunosuppressive activity. Specifically, observations of proliferative findings in the 6-month rat study, genetic polyploidy, and potential prolactin elevation were identified as potential tumorigenic liabilities beyond the risk from immunosuppression, which could be informed by 2-year rat data. The negative tumor outcome mitigated these concerns, although there is recognition that these concerns might have been adequately de-risked by investigative studies to reduce the need for a 2-year rat study. It is recognized that the primary human risk from immunosuppression would not be further informed by a 2-year rat study (Bugelski et al., 2010). The counterview is that the tumorigenic risk of compound #123 would be disclosed with appropriate labeling regardless of the tumor outcome from a 2-year rat study, or whether potential off-target tumorigenic risk is prospectively recognized or not.

### Weight of Evidence (WoE) factors

The 2013 RND described the WoE factors that should be addressed in preparing the carcinogenicity assessment documents for the PES. These factors were in part informed by the retrospective analyses from Sistare et al. (2011) and Van der Laan et al. (2016a, 2016b), where the pharmacology, histopathology, genotoxicity, and endocrine endpoints were considered key attributes in assessing the carcinogenic potential of pharmaceuticals in rats. The RND (ICH, 2013) and the finalized S1B(R1) Addendum (ICH, 2022) incorporated these endpoints and expanded the WoE factors to include consideration of the metabolic profile, secondary pharmacology, and immunotoxicity. The WoE assessment took into account all of these factors, but the relative importance of each factor varied depending on the compound being assessed. While a low level of concern for all factors was generally considered supportive of not conducting a 2-year rat study, a clear finding of high concern for any one factor (e.g., multi-tissue hyperplasia related to pharmacology) that cannot be resolved by other investigative approaches may necessitate the need for a 2-year rat study to address that uncertainty. More commonly, cause-for-concern was identified for multiple WoE factors. The DRAs were more likely than sponsors to conclude that a 2-year rat study was appropriate in such cases (e.g., Tables 8, 10). The attributes of each WoE factor and their relative contribution to an integrated assessment of carcinogenic risk and the need for 2-year rat data is captured in the decisional framework depicted in Figure 2. This framework is incorporated into the ICH S1B(R1) addendum as an aid to determine whether the human carcinogenic potential of an investigational pharmaceutical is likely, unlikely, or uncertain. These ‘risk categories’ described in the addendum correspond to Categories 1, 3a/3b, and 2 as described in this report, and are accompanied by regulatory recommendations regarding the potential added value of conducting a 2-year rat study.

The availability of an established profile of other compound(s) in a drug class often contributed substantially to assessing human carcinogenic risk and was particularly relevant to informing the target biology WoE factor. Such information is limited or absent for compounds directed toward novel drug targets which presents a knowledge gap and increases uncertainty when assessing human carcinogenic risk. The PES dataset includes a total of 12 compounds with novel drug targets, of which 6 cases were designated as Category 3a or 3b (#s 103, 130, 118, 137, 136, 131), and in two cases by unanimous decision (#s 103, 130). In case #130, a cause for carcinogenic concern was not identified regarding drug target biology or compound selectivity, and no proliferative changes in any organs or tissues were observed at a high multiple of exposure in the 6-month study in rats (a pharmacologically relevant species). The high (54x) exposure multiple in this case provided additional assurance that modulation of the drug target at more clinically relevant drug concentrations would be highly unlikely to present a carcinogenic risk. In case #103, the sponsor provided results of a 2-year rat study from a comparable but discontinued compound which indicated a lack of tumorigenic potential from modulation of the pharmacological target after long-term exposure, in addition to no cause-for-concern identified from other WoE factors. The 2-year rat study yielded a negative tumor outcome for both these cases, in confirmation of the Category 3b categorization based on the WoE approach. Of note, for both these compounds, additional evidence was provided that supported a conclusion of no cause-for-concern regarding target biology, which successfully compensated for the lack of precedent for the drug class. A high exposure multiple in the 6-month toxicology study and availability of relevant 2-year rat carcinogenicity data with other compound(s) are only two examples of meeting a higher evidentiary standard that may lend further support for a using a WoE approach for compounds with a novel target. Other sources of data may also be applicable, which would likely vary by specific attributes of the compound and target, and it would be the sponsor’s obligation to justify the type and scope of evidence appropriate to support a WoE approach for novel targets.

## Conclusion

The ICH S1 PES was undertaken by the ICH S1B(R1) EWG to address the hypothesis that, for some pharmaceuticals, a WoE assessment may be sufficient to predict the outcome and value of the 2-year rat carcinogenicity study for assessing human carcinogenic risk in the absence of conducting a 2-year rat study. An additional objective of the PES was to assess the regulatory feasibility of a WoE approach by evaluating concordance among regulators from five ICH regions following independent assessment of CADs and FSR summaries, as submitted by the sponsors.

The outcome of the PES suggests that, for some investigational pharmaceuticals, a WoE approach can be used to determine if a 2-year rat study adds value to the human carcinogenic risk assessment, and the ICH S1B guideline can be expanded to include recommendations supporting a WoE approach. Based on the number of DRA-designated unanimous Category 3a and 3b cases, approximately 27% of 2-year rat studies could be omitted and a WoE approach could instead be relied upon to characterize human carcinogenic risk. The WoE attributes that define this subset of cases included target biology of the parent compound and major human metabolites that is well characterized and not associated with cellular pathways known to be involved with human cancer development, secondary pharmacology that does not identify concerns for off-target potential, chronic toxicity studies that indicate no hyperplastic, hypertrophic, atypical cellular alterations, or degenerative/regenerative changes without adequate explanation of pathogenesis or human relevance, no alterations of endocrine or reproductive organs that are not adequately explained in relation to potential human relevance, no evidence of genotoxic potential, and no evidence of immune modulation or immunotoxicity based on target biology and repeat-dose toxicology studies.

The numerous cases where the sponsor and the DRAs independently and unanimously arrived at the same CAD categorization illustrate that harmonized decisions on the necessity of a 2-year rat study are feasible. Nonetheless, conclusions can and are expected to differ on occasion given the complexity of integrating risk information from multiple WoE factors. As the ICH S1B(R1) Addendum is implemented across the ICH regions, it will be important to monitor how sponsors will apply the recommendations in the Addendum and track the extent of DRA alignment in their recommendations to the industry regarding the acceptance of a WoE approach *in lieu* of a 2-year rat study. Implementation of this integrative approach is anticipated to reduce the use of animals in accordance with the 3R (reduce/refine/replace) principles and ideally shift resources to focus on generating more scientific mechanism-based carcinogenicity assessments, while continuing to promote safe and ethical development of new pharmaceuticals.

## Data Availability

The original contributions presented in the study are included in the article, further inquiries can be directed to the corresponding author.

## Footnotes

1 Case #140 was designated as Category 3b by the sponsor and Category 2 by the DRAs. The sponsor called the 2-year rat study positive based on a statistically significant trend in urinary bladder papilloma, whereas DRAs considered the outcome equivocal due to the lack of a dose-response and the marginal increase above historical controls.
